# Integrating new knowledge into practice: An evaluation study on a continuing education for Swedish child health nurses on non‐synostotic plagiocephaly

**DOI:** 10.1002/nop2.142

**Published:** 2018-04-17

**Authors:** Freda Lennartsson, Per Nordin, Beth Maina Ahlberg

**Affiliations:** ^1^ Institutionen for kliniska vetenskaper Avdelning för pediatrik Göteborgs universitet Göteborg Sweden; ^2^ Skaraborgsinstitutet Skövde Sweden

**Keywords:** child health care, education, non‐synostotic plagiocephaly, nurses’ instruction, parents, prevention, SIDS

## Abstract

**Aim:**

The aim of this study was to assess what knowledge on non‐synostotic plagiocephaly prevention and reversal intervention and control group nurses imparted to parents and parents integrated in infant care.

**Design:**

A group of nurses participated in a continuing education on non‐synostotic plagiocephaly in one‐to‐one or small group sessions at their workplace and received guidelines to follow. An evaluation was conducted with 35 intervention group and 15 control group nurses by asking them two open‐ended questions 1 year later; 181 intervention group and 90 control group parents were also asked two open‐ended questions and to rate their infant's head shape.

**Methods:**

Data were collected during the year 2013. Qualitative content analysis and case‐by‐case analysis were conducted using a process‐oriented approach.

**Results:**

Intervention group nurses imparted both regular and newly introduced knowledge on positioning strategies to parents. Intervention group parents who perceived severe cranial asymmetry when infants were 3–4 months old reported implementing regular and new infant positioning recommendations.

## 1 INTRODUCTION

Non‐synostotic plagiocephaly (NSP) is acquired cranial asymmetry that develops from external pressure on the infant's cranium. The prevalence seems to have increased after the campaign in the early 1990s recommending parents to place infants supine instead of prone for sleep to prevent sudden infant death syndrome (SIDS) (Biggs, 2004). While SIDS prevention must always be the priority, the supine sleep recommendation should not be confused with how to position infants when they are awake.

In a Canadian cohort study, an estimated 47% of infants had some degree of NSP at 7–12 weeks of age (Mawji, Vollman, Hatfield, McNeil, & Sauve, 2013). The most commonly reported risk factors are firstborn, male, limited neck rotation or preference in head position, supine sleep position, lower level of activity and lack of tummy time (De Bock, Braun, & Renz‐Polster, 2017). The prevalence seems to peak at 4 months and then decreases as infants grow older (Hutchison, Hutchison, Thompson, & Mitchell, 2004).

## 2 BACKGROUND

Although NSP might correct itself as an infant grows older and increased mobility relieves pressure on the cranium, this is not always the case. In a study from New Zealand of 129 children diagnosed in infancy with NSP and whose parents had been given information on counter‐positioning strategies, 39% had not reverted to the normal range of symmetry at mean age of 4 years and 13% of parents reported still being somewhat or very concerned (Hutchison, Stewart, & Mitchell, 2011). These results indicated that NSP did not always improve when information on counter‐positioning strategies was given to parents—not even when concerned parents were given information. In a study comparing developmental outcomes at 36 months of age in 224 preschool children with a history of NSP and 231 without, the children with a history of NSP scored lower on all of the Bayley Scales of Infant and Toddler Development, Third Edition. Furthermore, control group children who were later rated by paediatricians to have at least mild NSP also scored lower than unaffected controls (Collett et al., 2013). Although no cause–effect relationship was implied, results of this study indicate that even mild NSP might have implications. Since the course for NSP is difficult to predict, prevention efforts are warranted.

In a Canadian study investigating risk factors for NSP, researchers strongly advise that the recommendation to vary infants’ head position be communicated to parents well before the 2‐month well‐child clinic visit due to the high prevalence of NSP in infants aged 7–12 weeks (Mawji et al., 2014). A teaching programme to promote safe sleeping and also prevent NSP was developed for nurse practitioners in South Carolina, USA (Miller, Johnson, Duggan, & Behm, 2011). This programme consisted of a short handout that quickly explains the reasoning behind supine sleep positioning and the importance of this sleeping position for SIDS prevention. These researchers strongly advise healthcare professionals to stress the importance of the supine sleep position in preventing SIDS while also teaching parents techniques to minimize NSP.

From 2003 to late 2013, the Swedish Board of Health and Welfare's sleep position recommendations to prevent NSP while infants sleep safely were place the infant supine, make sure that the infant is comfortably warm and can move, alternate the direction of the infant's head and use a pillow until the infant starts to turn over (Socialstyrelsen [The Swedish Board of Health and Welfare], 2006). Although the American Academy of Pediatrician's Task Force on SIDS considers pillow use in the crib a suffocation risk and national guidelines in the United States, Canada and New Zealand, for example, warn against pillow use in the crib (Ministry of Health New Zealand, 2015; Moon, 2011; Public Health Agency in Canada, 2011), in Sweden, the use of an infant‐adapted pillow is considered safe until the infant starts turning over. The incidence of SIDS in Sweden did not rise when the pillow recommendation was introduced and did not decrease when the pillow recommendation was removed in late 2013. The incidence of SIDS per 1 000 live births in Sweden was 0.21 in 2002, 0.18 in 2013 and 0.14 in 2014 (Socialstyrelsen [The Swedish Board of Health and Welfare], 2015), that is, before, during and after the pillow recommendation respectively. In an international comparison of SIDS incidence in 13 countries from 1990 to 2005, the incidence in Sweden was very low (Hauck & Tanabe, 2008). This indicates that the Swedish pillow recommendation did not compromise infant safety. However, there was scarce evidence that pillow use helped prevent NSP, and the Swedish pillow recommendation was therefore removed in late 2013 (Socialstyrelsen [The Swedish Board of Health and Welfare], 2013). The national sleep position recommendations in 2018 to prevent NSP while infants sleep safely includes the following: place the infant supine, make sure that the infant is comfortably warm and can move and alternate the direction of the infant's head.

Sweden has a national child healthcare programme and nurses at the child health clinics are the primary healthcare providers responsible for monitoring infants’ growth and development. Nearly all infants in Sweden attend the child health clinics, providing an ideal venue for both monitoring infant cranial shape and imparting knowledge to parents. The nurses have the responsibility of informing parents of newborns about the Swedish Board of Health and Welfare's current SIDS prevention recommendations and infant sleep position recommendations. At the time of this study, the national child health care programme had their own recommendation—that parents place infants prone under surveillance when infants are awake, a recommendation that is now included in the Swedish Board of Health and Welfare's recommendations (Socialstyrelsen [The Swedish Board of Health and Welfare], 2013).

In the late 1990s, child health nurses in the Skaraborg County began noticing that quite a number of infants coming for well‐child visits had obvious NSP. In some cases, the asymmetry had become severe before anyone reacted. The nurses had no education on NSP. Reversal advice consisted of repeating prevention recommendations and encouraging parents to increase “tummy time,” time infants spend lying prone when awake, which were the child health programme's own recommendations. However, on subsequent visits, many parents explained that their infants refused tummy time; and infants’ cranial asymmetry had not decreased. A prevention project was thus initiated in 2008 in an attempt to improve practice and the first author of this study became the project leader. Following a literature search on prevention practices, guidelines for nurses were developed, tested in a pilot study and revised (Lennartsson, 2011a, 2011b). A short continuing education on NSP, which included the revised guidelines, was subsequently developed for the nurses. Then, an intervention study with a two‐armed design was conducted. Firstly, intervention group nurses were educated about NSP. Secondly, a longitudinal clinical intervention was conducted. The present study was conducted alongside the intervention study.

The aim of this study was to assess what knowledge on non‐synostotic plagiocephaly prevention and reversal intervention and control group nurses imparted to parents and parents integrated in infant care. The assumption was that educating child health nurses about NSP would lead to new knowledge being integrated into practice while still promoting safe infant positioning practices. Two research questions were: What did the nurses report they informed parents about NSP prevention and reversal? What did the parents report they had done to prevent their infants from developing NSP and to reverse NSP if it developed?

## 3 THE STUDY

### 3.1 Design

**Figure 1:**
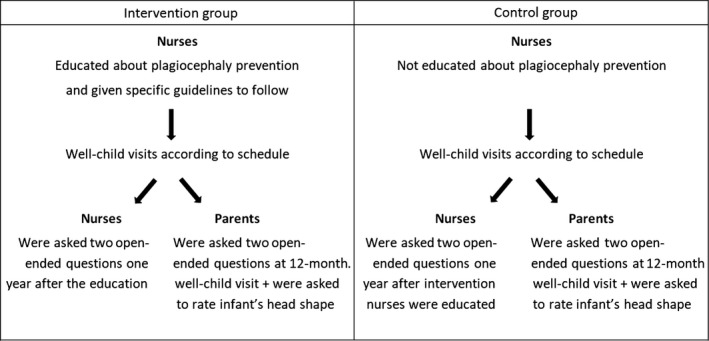
Design of the study

Thirty‐five nurses (intervention group) participated in a short education on non‐synostotic plagiocephaly led by the project leader. The education took approximately 1½ hr and was conducted in one‐to‐one or small group sessions at each nurse's workplace. Sessions were conducted in January 2012. One year later, intervention and control group nurses were asked two open‐ended questions. Intervention and control group parents were similarly asked two open‐ended questions in conjunction with the 1‐year well‐child visits. Parents were also asked to rate their infant's head shape. Figure 1 illustrates the design of the study. This is an evaluation study of the continuing education. Since this study was conducted alongside the intervention, it has the same prospective cohort design.

### 3.2 Methodology

#### 3.2.1 The nurse education

The nurse education included information on: how NSP develops; risk factors; which infants are especially vulnerable; how to assess cranial shape; how to differentiate NSP from craniosynostosis; the national recommendations; the child healthcare programme recommendations; further recommendations for parents on how to prevent NSP; recommendations for parents on how to reverse incipient asymmetry; and infant safety aspects. Nurses were asked to begin imparting the knowledge to parents of newborns by 2 weeks postpartum, to consult a physician when cases did not improve within 2 months and to promptly consult a physician if craniosynostosis was suspected. Nurses were given a folder containing the guidelines for nurses; the Severity Assessment for Brachycephaly and the Severity Assessment for Plagiocephaly (Cranial Technologies Inc, 2002a, 2002b) to guide them in carrying out asymmetry cranial assessments; a leaflet to distribute to parents; and a brochure with suggestions to make tummy time more acceptable for infants (Öhman, 2007).

#### 3.2.2 Setting and group allocation

The study was conducted at 26 of the 32 child health clinics in Skaraborg County. Group allocation in this study was the same as group allocation in the intervention study. Group allocation was determined by nurses’ exposure to the project prior to the intervention. Thus, if any nurse at a clinic had participated in the pilot study conducted in 2008 and/or had attended the group lecture on NSP in 2010, all nurses at that clinic were placed in the intervention group. This was done to avoid the so‐called spillover effect where nurses who were exposed to new information might influence colleagues. Nurses at clinics where no one had participated in the pilot study or attended the above‐mentioned lecture were placed in the control group. Infants and parents were placed in the same group as their nurses.

#### 3.2.3 Data collection and participants

Nurses and parents who participated in the clinical intervention were asked to answer two open‐ended questions by filling in a form in 2013. The form was sent to 53 nurses (36 intervention group and 17 control group) 1 year after the nurse education. All were public health or paediatric nurse specialists. Fifty nurses responded (35 intervention group and 15 control group) and three nurses failed to respond. Two hundred and seventy‐one parents (181 intervention group and 90 control group), one parent for each infant, were asked to fill in a form in conjunction with the 1‐year well‐child visits. When families missed their clinic appointment, we asked parents for permission to make a home visit. All parents filled in a form.

#### 3.2.4 Materials

The form for nurses included two open‐ended questions with the suggestion to list responses and a space at the end for them to comment. The suggestion to write a list was included in the hope it could encourage multiple responses. The open‐ended questions were: What advice do you give parents of newborns on prevention of cranial asymmetry? When you discover an asymmetry and what do you advise parents? The space for comments was included to elicit narratives. The parent form began with a section asking if they thought the back of their child's head was flattened and if it was skewed at 3–4 months of age and at 12 months of age. There were three alternatives — no, a little and a lot, which correspond with none, noticeable and severe respectively. Secondly, there were two open‐ended questions including: What did you do to prevent your child from developing cranial asymmetry? If your child nevertheless developed cranial asymmetry, what did you do? At the end of the form, there was space for comments.

### 3.3 Analysis

**Figure 2:**
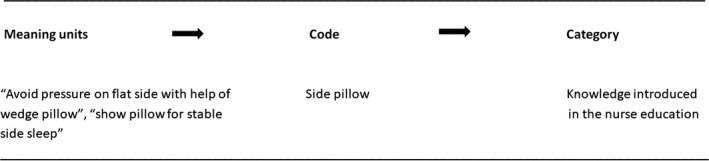
Example of the coding scheme

A qualitative content analysis with a process‐oriented approach (Maxwell, 2010) was conducted using the framework method (Gale, Heath, Cameron, Rashid, & Redwood, 2013). The analysis therefore followed information flow from nurses’ sources of knowledge → nurses’ recommendations to parents → parents’ infant care practices. A coding scheme was developed before and during the analysis. Responses were reviewed carefully. Meaning units were identified and codes were agreed on after discussion among the three co‐authors. Figure 2 is an example of the coding scheme.

**Figure 3:**
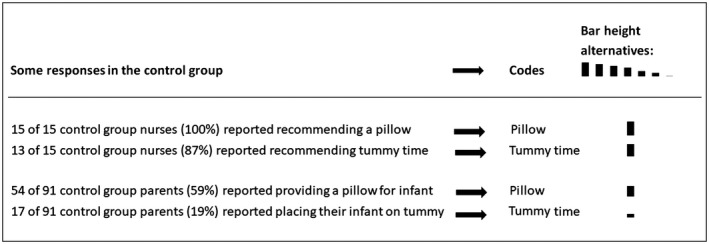
Illustration of how codes were ranked in descending order within a group and assigned bar heights

Responses were coded. Then, categories of knowledge were identified and each code was given a category of knowledge denotation. Coded responses were subsequently organized in a matrix to enable group comparisons. The matrix output is the defining feature of the framework method. The matrix output of rows and columns and “cells” of summarized data provides a structure into which the researcher can systematically reduce the data to analyze it by case and by code in a way that can support answering the research questions (Gale et al., 2013). The codes in each nurse and parent column were arranged in descending order of importance and given bars where the height of each bar represents the relative importance of a code based on its’ occurrence. Figure 3 illustrates how the codes were ranked in a column and assigned bar heights. Rankings and bar heights are semi‐quantitative components that are used to provide meaningful visual patterns in the matrix (Groenland, 2014). As Maxwell argues, counting the number of instances when participants in a qualitative study perform a particular action can be interpreted in process terms (Maxwell, 2010). The semi‐quantitative components were included to enable group comparisons in our process‐oriented approach. Readers can follow what knowledge was put into practice in the two groups by studying the matrix. Separation of intervention and control groups depicts the different exposures to sources of knowledge in the two nurse groups. Arrangement of codes in nurses’ columns illustrates occurrences and sources of knowledge of specific recommendations. Arrangement of codes in parents’ columns illustrates occurrences and sources of knowledge of specific infant care practices. This systematic structure provides a comparative overview which helps answer our research questions.

A qualitative case‐by‐case analysis, including cases parents perceived as severe at 3–4 months, was conducted with a process‐oriented approach. Analysis followed the process from nurse's sources of knowledge → parent's perceptions of severe asymmetry at 3–4 months → nurse's recommendations to parents → parent's infant care practices → parent's perceptions of asymmetry at 12 months. The process was examined step‐by‐step for each case to gain an understanding of what might contribute to reversal.

Table 1 illustrates the design of the two analyses.

**Table 1:**
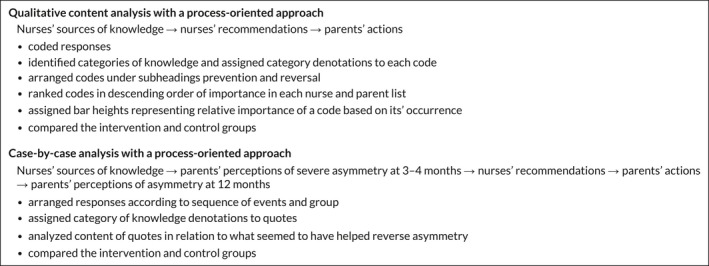
Design of the analyses

### 3.4 Rigour

Using two methods of analysis provides a more complete understanding of the data and strengthens the credibility of the results. The qualitative content analysis provides an understanding of what knowledge was implemented in practice on a group level and the qualitative case‐by‐case analysis provides an understanding of what knowledge was implemented in practice on an individual level. The arrangement of codes in the matrix of the content analysis, quotes in the case‐by‐case analysis and category denotations in both provides transparency.

### 3.5 Ethics

All recommendations, including pillow recommendations, were in line with official Swedish SIDS and infant positioning guidelines (Wennergren et al., 2015). Parents provided written informed consent. Intervention group nurses were asked to consult a physician if cases did not improve within 2 months and to promptly consult a physician if craniosynostosis was suspected. If severe asymmetry was observed in conjunction with data collection, the parents were offered an appointment to the project leader for in‐depth advice. When all data were collected, each control group nurse was offered the opportunity to participate in the nurse education at their own clinic. The Regional Ethical Review Board in Gothenburg approved the study (Dnr T887‐12).

## 4 RESULTS

In the qualitative content analysis, codes are arranged under the subheadings prevention and reversal (Table 2). Most codes pertain to infant positioning. Examples of codes include pillow, tummy time, vary position, carry, sit, alternate head position and minimize bouncer time. Four categories of knowledge identified among codes include: 1) knowledge from the official national recommendations (small caps); 2) knowledge from the child health programme (regular print); 3) knowledge introduced in the education (in bold); and 4) incorrect knowledge (in red). The first three categories are sources of knowledge conducive to NSP prevention and reversal. The fourth category pertains to responses that are not conducive to NSP prevention and reversal or that are unsafe. Each code is denoted by the category of knowledge from which it originated. The codes in each nurse and parent column are arranged from most frequently to least frequently reported. The ranking corresponds with bar heights that go from tall to short.

**Table 2:**
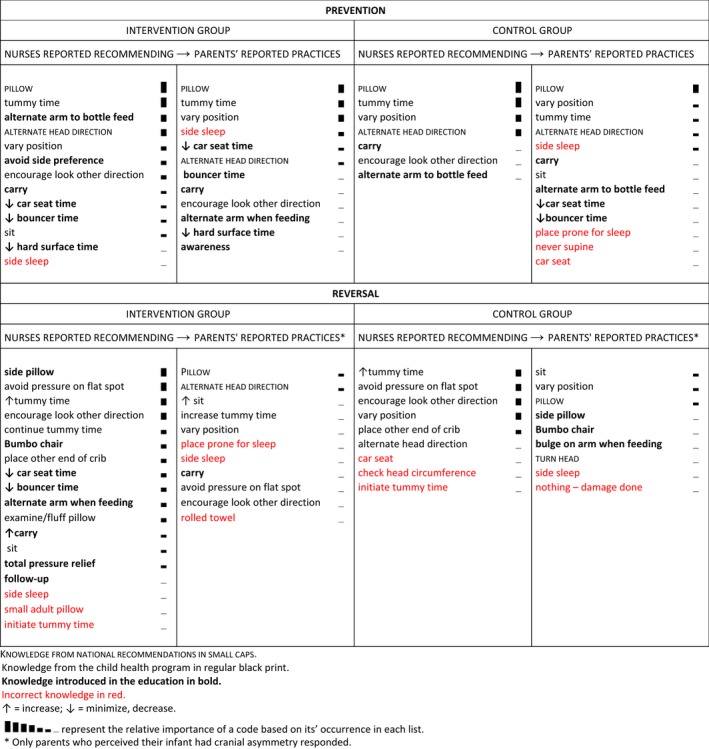
Qualitative content analysis exploring what knowledge from a continuing education for nurses on non‐synostotic plagiocephaly had been implemented in practice by comparing intervention and control group nurses’ and parents’ coded responses to open‐ended questions on prevention and reversal

As seen in the lists of codes in Table 2, nearly all nurses in both groups reported recommending pillows, an official national recommendation. Nurses in the control group reported recommending knowledge from the official national and the child health programme recommendations, considered “regular” knowledge, nearly as often as intervention group nurses. However, as could be expected, intervention group nurses reported recommending more “new” knowledge, that is, knowledge introduced through the education, than control group nurses. Most intervention group nurses reported recommending a side pillow, which is a wedge pillow for safe side sleep. The side pillow is considered new reversal knowledge. Many intervention group nurses reported recommending other new knowledge as well, including how to accomplish occipital pressure relief when infants are awake, asleep and being fed. However, the relative frequency of these positioning recommendations was low.

Parents’ responses were generally short. Parents who did not perceive any cranial asymmetry in their infants naturally had no reversal efforts to report. Intervention and control parents’ lists of codes were similar. Parents in both groups mostly reported regular knowledge on infant positioning. Pillow was their most common response. Yet, as seen in the case‐by‐case analysis (Table 3), intervention group parents also reported incorporating new knowledge into infant care. Nurses’ and parents’ responses are quoted in the table. Quotes have category of knowledge denotations as above and portions of quotes that could not be sorted into any category are denoted in grey. What is striking in Table 3 is that at 12 months, most intervention group parents reported complete reversal of severe cranial asymmetry, while no control group parent did. We examined the nurses’ and parents’ responses to understand why the intervention group seemed successful in reversing severe cranial asymmetry. Nurses and parents reported repositioning strategies from the official national recommendations, child health programme recommendations and nurse education. This infers that severe cranial asymmetry reversed when regular and new recommendations were implemented in joint nurse–parent efforts.

**Table 3:**
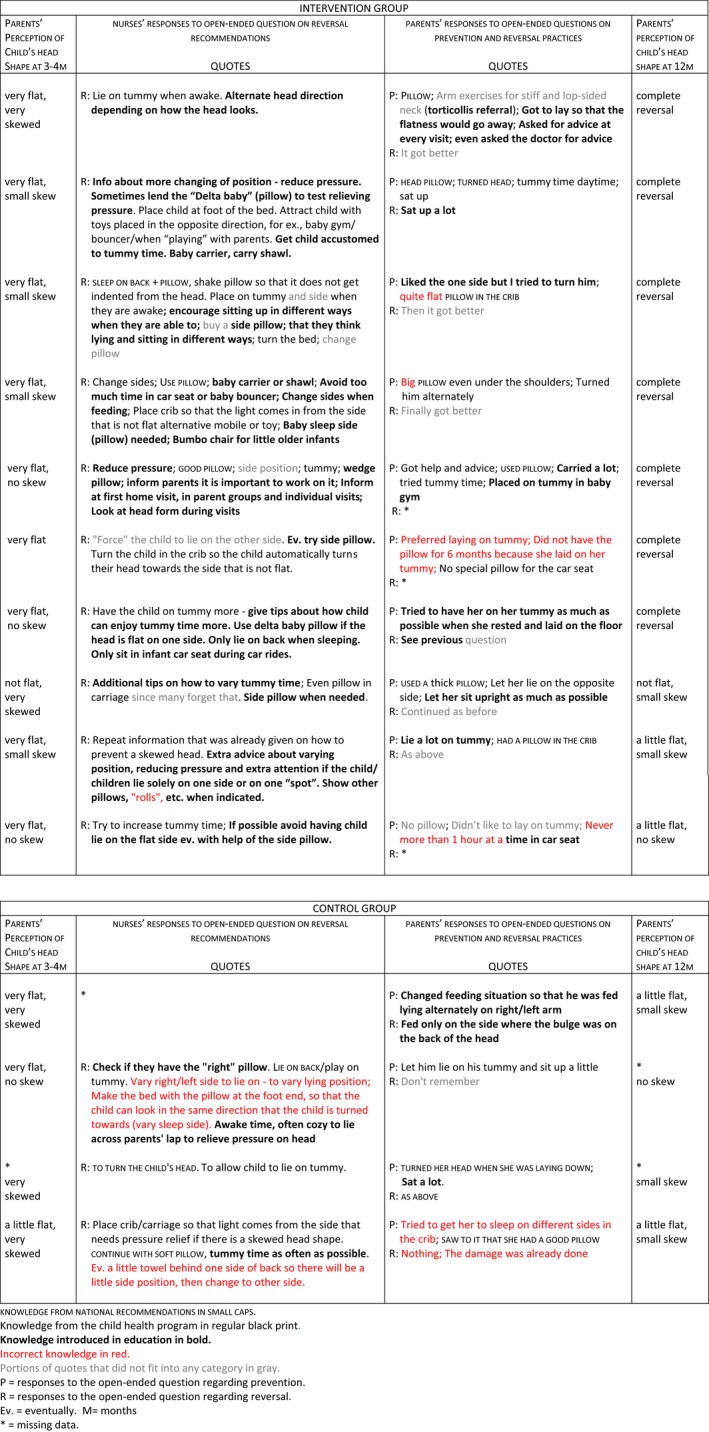
Qualitative case‐by‐case analysis exploring what knowledge from a continuing education for nurses on non‐synostotic plagiocephaly was implemented in practice and what seemed to have contributed to reversal

Several results have implications for infant safety. One is that most nurses and parents that reported *tummy time* did not report the safety details of *tummy time*—that infants are awake and under surveillance when they are placed prone. Another result with safety implications is that *side sleep*, a code seen in both nurses’ and parents’ lists, is a risk factor for SIDS (Alm et al., 2006). A third result with safety implications is that some parents in both groups reported *placed prone for sleep,* which is also a risk factor for SIDS. Some parents provided explanations as to why they placed their infant prone for sleep (Table 4). Parents’ explanations include:

**Table 4:** Parents’ explanations for placing their infant prone for sleep

Intervention group “Tried to change sides as long as I could but he chose to lie on his tummy early and was strong and changed and turned his head himself. He was born with a nice head. P.s. never slept on his back.” “I allowed her to sleep a lot on her tummy on my chest and even lie on her tummy when she was awake.” “She slept on my tummy the first 3 months. Did not want to sleep by herself on her back so we didn't have to do anything special to counteract that.” Control group “He always slept on his tummy. He didn't like to sleep on his back.” “Nothing special actually. He refused to sleep on his back. He slept a lot on his tummy. I believe that made a difference.”

…I tried…but he chose to lie on his tummy early and was strong…

I allowed her to sleep a lot on her tummy on my chest…

She slept on my tummy the first 3 months. Did not want to sleep by herself on her back…

…always slept on his tummy. He didn't like to sleep on his back.

…He refused to sleep on his back…

## 5 DISCUSSION

The aim of the study was to assess what knowledge on non‐synostotic plagiocephaly prevention and reversal intervention and control group nurses imparted to parents and parents integrated in infant care. Our assumption was that educating child health nurses about NSP would lead to new knowledge being integrated into practice, while still promoting safe infant positioning practices. The major findings of the study are: Intervention group nurses reported recommending both regular and new positioning strategies to parents regarding NSP prevention and reversal, including ways to accomplish occipital pressure relief when infants are awake, are asleep and are being fed, and intervention group parents who perceived severe cranial asymmetry at 3–4 months reported implementing both regular and new positioning strategies in their infant care. These findings answered our research questions. However, in some cases, safety information was lost during the process. Another important finding is that of the parents who perceived severe cranial asymmetry at 3–4 months, only intervention group parents reported complete reversal at 12 months. Although not directly comparable, it is worth noting that except for two infants, parent‐perceived cranial shape at 12 months in the case‐by‐case analysis corresponds with the infant's 12‐month cranial asymmetry assessment in the clinical intervention—assessments conducted by trained assessors blinded to group. Together, these findings infer that intervention group nurses provided tailored reversal advice to parents when needed and that intervention group nurses’ and parents’ joint efforts in following both regular and new recommendations seemed to work in reversing NSP.

The nurses had recognized from their own work‐related experience that NSP was a problem and that their efforts to reverse NSP had often been futile. The idea to educate the nurses on NSP prevention came from one of the child health nurses, who became the project leader. Being a colleague who had worked in the child health programme for over 18 years, the project leader was trusted as an experienced insider. The nurses accepted the project leader as one of them and for this reason, the project leader was not perceived as a threat when suggesting the education. The nurses were motivated to learn from a peer in hopes of improving practice.

There are similarities between our study and a qualitative nursing study from the UK (Simons, Lathlean, & Squire, 2008). That study was similarly conducted alongside an intervention study where nurses were allocated to an intervention group receiving an education or a control group. The researchers found that nurses in the intervention group actively applied their new knowledge and that nurses in the control group aimed for positive change using their existing skills and experience. Their findings regarding how the two nurse groups applied knowledge are consistent with our findings.

In a qualitative study using focus group discussion and semi‐structured interviews in Australia, parents’ views of NSP prevention were examined. (Martiniuk, Jacob, Faruqui, & Yu, 2016). The researchers reported that some parents were more concerned about preventing NSP than SIDS because NSP was more of a reality. They found that once concern or discovery of NSP occurred, the majority of parents stopped adhering to the country's SIDS guidelines on safe infant sleep, including placing infants supine for sleep and not providing pillows when infants sleep. In contrast, we did not observe parent incompliance to SIDS guidelines in our study. Few parents in our study reported placing their infants in unsafe sleep positions. Parents who did report placing their infants prone for sleep provided explanations which indicate *unawareness* of SIDS guidelines, not incompliance (Table 4); however, we could not probe since we did not interview. Reports regarding pillows were not incompliance to SIDS guidelines either, since pillows were included in official Swedish national recommendations at the time of the study.

Pillow was the most common nurse and parent prevention response in our study. However, after our study was conducted, the Swedish Board of Health and Welfare removed the pillow recommendation in December 2013 because there was little evidence supporting pillow use in NSP prevention (Socialstyrelsen [The Swedish Board of Health and Welfare], 2013; Wennergren et al., 2015). Removal of the pillow recommendation from the national recommendations left both nurses and parents in Sweden without their most frequent NSP prevention strategy. Consequently, the focus of our NSP prevention efforts changed. The focus is now on alternating the direction parents place a neonate's head for sleep and tummy time under surveillance when infants are awake. However, changed recommendations are a potential source of confusion for parents, especially when recommendations change between the births of their children. It is therefore the job of the child health nurse to always keep up to date on national recommendations and inform parents about them.

Tummy time when infants are awake and under surveillance provides complete occipital pressure relief while insuring infant safety. In a cross‐sectional survey in North Carolina, USA, examining parental practices related to infant positioning and infant‐positional devices to identify predictors of cranial asymmetry, parents of children who did not develop NSP reported more tummy time than parents of children who did develop NSP (Zachry, Nolan, Hand, & Klemm, 2017). In our study, tummy time was the second most common prevention response provided by nurses in both groups and by intervention group parents. In contrast, few control group parents reported tummy time as a prevention practice, although tummy time was a regular recommendation, not a new one. This finding implies that our intervention helped more parents understand the importance of tummy time and thereby increased compliance. Tummy time under surveillance is time consuming and parents of infants are busy, so understanding this time‐consuming recommendation is important both for infant safety and head shape.

Parents’ understanding of tummy time recommendations is important if we want to improve practice. In a study investigating overcoming barriers of a parent education intervention on tummy time when Latino parents had limited English proficiency, parents’ knowledge of tummy time increased when educational materials were translated into Spanish and trained bilingual assistants delivered the intervention (Nitsos, Estrada, & Messias, 2017). We could help increase the understanding of NSP prevention recommendations for immigrant parents with limited Swedish proficiency if we translate educational materials into their languages and if nurses use interpreters when counselling parents.

### 5.1 Reflexivity

Our process‐oriented approach contributes to understanding of how data are linked. Information flow in the two groups can be followed in the matrix of the qualitative content analysis and in the case‐by‐case analysis. In the case‐by‐case analysis, the arrangement of parents’ perceptions and nurses’ and parents’ quotes in sequential order enable the process from source of knowledge to parents’ perceptions of cranial asymmetry in 12‐month‐old infants to be followed. This sheds some understanding on what recommendations and infant care practices helped reverse NSP, understanding that would have been missed otherwise.

The semi‐quantitative components in the content analysis enable comparisons within and between groups while still retaining connection to all respondents. In this way, we were able to identify similarities and differences between nurses and parents and between intervention and control groups, similarities and differences that would have been missed otherwise.

#### 5.1.1 Limitations

Although response rate was high, asking participants to answer two open‐ended questions by filling in a form precluded the opportunity for follow‐up, common when interviewing face‐to‐face. Parent responses were generally short, and we could not probe because we were not interviewing them. Using a form instead of interviewing and lack of follow‐up are considered limitations of the study. Using the suggestion “write a list” hindered parents from providing expansive qualitative responses and is considered another limitation. A further limitation of the study is that we did not collect data on socio‐demographic factors. Some short parental responses could have been due to limited Swedish proficiency.

Short parental responses could also have been due to decreased recall because of time lapse. While nurses used their knowledge in daily practice, parents of the 12‐month‐old infants had to think back about 9 months. Data were collected when infants were 12 months old for the following reasons: One year after the education was deemed an appropriate time to follow‐up what nurses had incorporated into practice; it was convenient to distribute forms to parents in conjunction with the planned 1‐year visit when we were collecting other data; we had distributed checklists to these parents in conjunction with two previous visits, so this familiar procedure helped minimize parent non‐response; and parents’ perception of their infant's head shape at 12 months was needed for the analysis. Short parental responses could also have been due to parents rushing to fill in a form while visiting the clinic and/or parents might have been worried and unfocused on answering questions because the 12‐month well‐child visit was also vaccination day. The time lapse and collecting data from parents in conjunction with 12‐month well‐child visits are also considered limitations of the study. On the other hand, it is possible that parents did not have more to report. Most parents who reported cranial asymmetry at 3–4 months did report some reversal efforts.

## 6 CONCLUSIONS

Educating child health nurses about NSP and providing them with specific guidelines to follow contributed to new knowledge on infant positioning being implemented in practice. Our findings infer that the joint efforts of nurses and parents implementing regular and new knowledge in practice seemed to be effective in reversing NSP. However, sometimes safety aspects did not get through to parents. Parents’ understanding of recommendations is important for infant safety and parent compliance, so nurses need to tailor information when counselling parents.

The continuing education and guidelines designed for the nurses in the intervention need more emphasis on occipital pressure relief when infants are awake, because NSP is widespread today and the pillow recommendation has been removed. Tummy time under surveillance provides total occipital pressure relief; however, this is time consuming. Furthermore, infants often protest unless tummy time is initiated early. Thus, parent understanding of tummy time recommendations is important for both infant safety and head shape.

Further research is needed to streamline the continuing education and guidelines for nurses, to investigate nurse and parent compliance and to evaluate what measurable effects the nurse education had on NSP prevention and reversal.

## CONFLICT OF INTEREST

All authors declare no conflicts of interest.

## AUTHOR CONTRIBUTIONS

All authors agree on the final version of the paper. FL had primary responsibility for designing the study, administrating data collection, analysis and writing the manuscript. PN supervised the design of the study and the analysis and critically appraised the content of the manuscript. BMA supervised the analysis and critically appraised the content of the manuscript.
